# Publisher Correction: Ancient DNA from Chalcolithic Israel reveals the role of population mixture in cultural transformation

**DOI:** 10.1038/s41467-018-06484-8

**Published:** 2018-09-20

**Authors:** Éadaoin Harney, Hila May, Dina Shalem, Nadin Rohland, Swapan Mallick, Iosif Lazaridis, Rachel Sarig, Kristin Stewardson, Susanne Nordenfelt, Nick Patterson, Israel Hershkovitz, David Reich

**Affiliations:** 1 Department of Organismic and Evolutionary Biology, Harvard University, Cambridge, MA 02138 USA; 2 Department of Genetics, Harvard Medical School, Boston, MA 02115 USA; 3 The Max Planck–Harvard Research Center for the Archaeoscience of the Ancient Mediterranean, Cambridge, MA 02138 USA; 4 Department of Anatomy and Anthropology, Sackler Faculty of Medicine, Tel Aviv University, Tel Aviv 6997801, Israel; 5 Shmunis Family Anthropology Institute, Dan David Center for Human Evolution and Biohistory Research, Sackler Faculty of Medicine, Steinhardt Natural History Museum, Tel Aviv University, Tel Aviv 6997801, Israel; 6 The Institute for Galilean Archaeology, Kinneret Academic College, Kinneret 15132, Israel; 7 Broad Institute of MIT and Harvard, Cambridge, 02142 MA USA; 8 Howard Hughes Medical Institute, Boston, MA 02115 USA; 9 The Maurice and Gabriela Goldschleger School of Dental Medicine, Sackler Faculty of Medicine, Tel Aviv University, Tel Aviv 6997801, Israel

Correction to: *Nature Communications* ; 10.1038/s41467-018-05649-9, published online 20 Aug 2018.

In the original version of this Article, references in the format ‘First author et al.’ were inappropriately deleted. These errors have been corrected in the PDF and HTML versions of the Article.

