# Supplementary material for: Ancient DNA from Chalcolithic Israel reveals the role of population mixture in cultural transformation
Source: Nat Commun. 2018 Aug 20;9:3336. doi: 10.1038/s41467-018-05649-9 (PMC6102297; doi:10.1038/s41467-018-05649-9)
Supplement: Supplementary file 1 — Supplementary Information [file 41467_2018_5649_MOESM1_ESM.pdf]

Supplementary Information

Ancient DNA from Chalcolithic Israel reveals the role of population mixture in cultural transformation

Harney et al.

Table of Contents

Supplementary Table 1: Background of samples yielding authentic ancient nuclear DNA. 2
Supplementary Table 2: Outgroup Population Abbreviations 3
Supplementary Figure 1: F_{ST} between West Eurasian populations 12,500 BCE to present. 4
Supplementary Note 1 – No detectable population structure in Levant Neolithic 5
Supplementary Note 2 – *f*-statistics... 7
Supplementary Note 3 – qpAdm Analyses 10
Supplementary Note 4 – Population Admixture into East Africa from the Levant 19
Supplementary References.. 20

Supplementary Table 1: Background of samples yielding authentic ancient nuclear DNA.

Sample ID	Skeletal codes	Skeletal element	No. of Libraries	Sex	mtDNA haplogroup	Y chrom. calls	MT consensus match rate	X-contamination estimate (for males with >200 X-chomosome SNPs)	Coverage	SNPs hit on autosomes	Notes
I0644	CHPK00003	petrous	1	F	H4	..	0.971	..	0.869	598616	
I1152	CHPK008	petrous	1	F	J2a2d	..	0.994	..	1.14	592889	
I1154	CHPK018	petrous	1	F	T2g1a	..	0.983	..	0.05	56279	
I1155	CHPK021	petrous	1	M	K1a	T1a1a*	0.962	(<200 SNPs)	0.09	92467	
I1160	CHPKL101B-005, CHPKL101B-011	petrous	2	M	N1a1b	T1a1a1b2	0.998	(<200 SNPs)	1.308	597303	
I1164	CHPKL104-002	petrous	1	F	J2a2d	..	0.992	..	0.362	312969	
I1165	CHPKL104-004	petrous	1	M	HV1a'b'c'	T1a1a1b2	0.997	0.00362	0.95	452444	
I1166	CHPKL104-014, CHPKL104-026	petrous	2	M	H	T1a1a	0.991	0.01	0.981	531821	Excluded from Analysis: Father or son of I1169
I1168	CHPKL104-034	petrous	1	F	T2+150	..	1	..	1.06	544900	
I1169	Pkinn CHPK: L105-012	petrous	5	F	J2a2d	..	0.985	..	8.29	1002682	
I1170	CHPKL105-030	petrous	1	M	T1a2	T1a1a	0.974	0.006322	0.67	467224	
I1171	CHPKL106-019	petrous	1	M	K	E1b1b1b2	0.984	(<200 SNPs)	0.26	224077	
I1172	CHPKL108B-024	petrous	1	M	K1a	T1a1a	0.982	(<200 SNPs)	0.12	122474	
I1177	CHPKL109M-013	petrous	1	F	N1b1	..	0.989	..	0.09	89076	
I1178	CHPKL109L-015	petrous	1	M	I6	T1a1a	0.984	0.005911	2.56	719331	
I1179	CHPKL109M-016	petrous	1	F	T2	..	0.992	..	0.513	399485	
I1180	CHPKL109M-028	petrous	1	M	T	T1a1a*	0.998	(<200 SNPs)	0.09	89537	Excluded from Analysis: 1st degree relative of I1179
I1181	CHPKL109M-029	petrous	1	F	T2	..	0.983	..	0.09	92732	
I1182	CHPKL109M-031	petrous	1	F	T1a+152	..	0.978	..	0.63	421398	
I1183_d	CHPKL109M-035	petrous	1	F	R0a	..	0.965	..	0.45	25171	Damage restricted
I1184	CHPKL1100-020	petrous	1	F	T1	..	0.958	..	0.41	325567	
I1187	CHPKL301N-001	petrous	1	M	U6d	T	0.976	<200 SNPs)	0.12	124452	

Detailed sample background data for each of the 22 samples from which we successfully obtained ancient DNA. Additionally, background information for all samples from Peqi'in that were screened is included in Supplementary Data 1. *Indicates that Y-chromosome haplogroup call should be interpreted with caution, due to low coverage data.

Supplementary Table 2: Outgroup Population Abbreviations

09	Ust_Ishim, Kostenki14, MA1, Han, Papuan, Onge, Chukchi, Karitiana, Mbuti
Haber	Ust_Ishim, Kostenki14, MA1, Han, Papuan, Ami, Chukchi, Karitiana, Mbuti, Switzerland_HG, EHG, WHG, CHG
A	Anatolia_N
B	Steppe_MLBA
C	CHG
D	Iran_LN
E	EHG
F	Armenia_EBA
G	SHG
I	Iberia_BA
J	Steppe_IA
L	Europe_LNBA
M	Europe_MNChL
N	Natufian
P	Steppe_EMBA
R	Armenia_MLBA
S	Switzerland_HG
T	Steppe_Eneolithic
W	WHG
Y	Iran_N
Z	Levant_ChL

These abbreviations are used to indicate the outgroups included in the "Right" population set during qpAdm analysis. Population abbreviations are concatenated in order to indicate all populations included in the "Right" set (e.g. the set 09NW includes all populations from the 09 set of populations (Ust_Ishim, Kostenki14, MA1, Han, Papuan, Onge, Chukchi, Karitiana and Mbuti) in addition to Natufian and WHG).

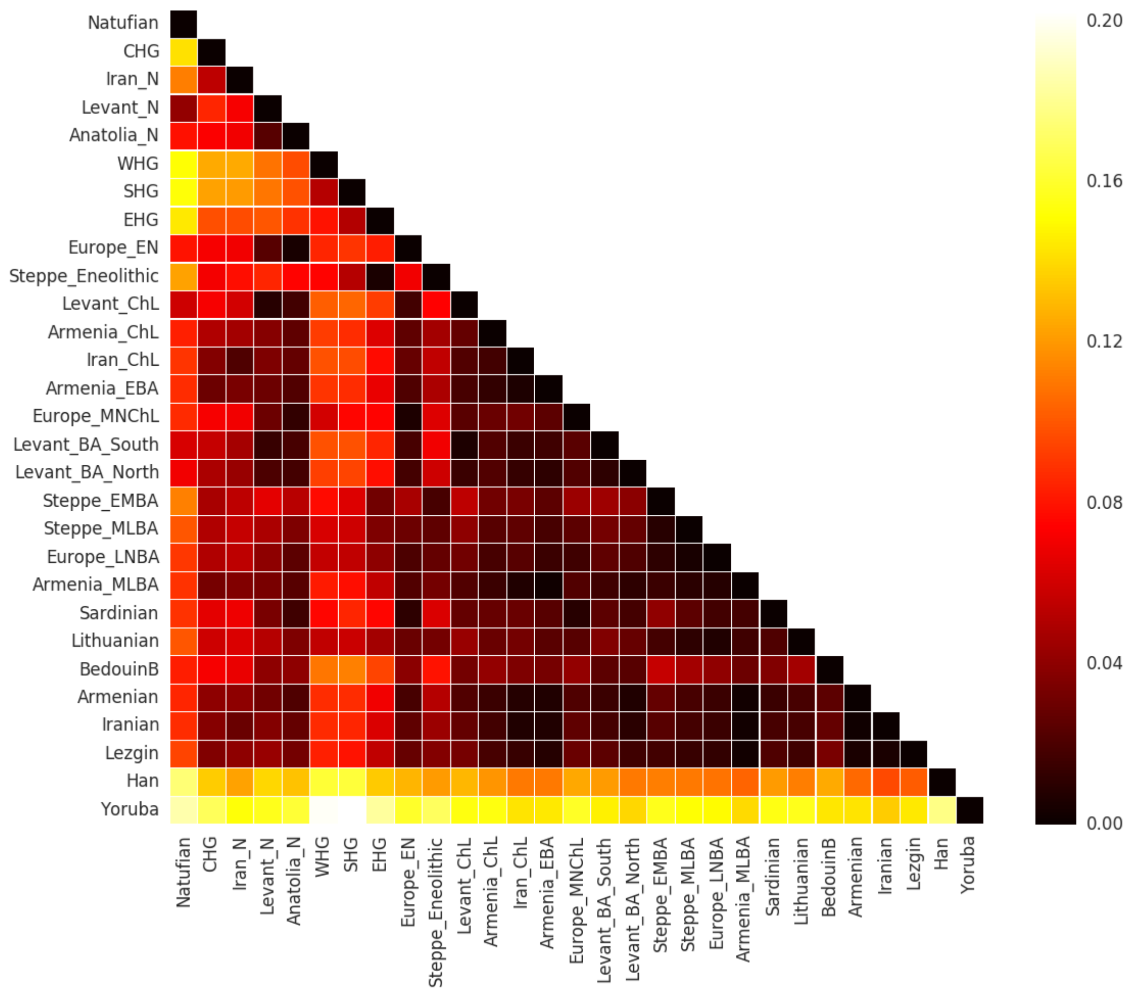

Supplementary Figure 1: F_{ST} between West Eurasian populations 12,500 BCE to present. Pairwise F_{ST} between 21 ancient and 8 present-day populations, arranged in approximate chronological order. As previously reported, between population differentiation is generally greater among more ancient populations (top and left), while more recent populations (bottom and right) exhibit less between population differentiation. The Levant_ChL population exhibits the greatest affinity to populations closely related to those from which it descends (ie. Levant_N, Anatolia_N, Iran_ChL) and later Bronze Age Levantine populations (i.e. Levant_BA_North and Levant_BA_South)

Supplementary Note 1 – No detectable population structure in Levant Neolithic

We explore the genetic composition of the Levant_N population in order to determine whether there is any evidence that population structure may be impacting our ability to model the ancestry of the Levant_ChL population. The Levant_N population was first reported in Lazaridis et al [1], where it was subjected to formal testing in order to determine whether it would be appropriate to consider all of the 13 Neolithic Levantine individuals that it includes to be a single, homogenous population for the purposes of genetic analyses. These tests did not detect structure in the Levant_N population to the limits of the statistical resolution used in that study, and they were therefore pooled for analysis. Nevertheless, there was suggestive evidence of potential heterogeneity among individuals during ADMIXTURE analysis (Figure 3b). Specifically, three individuals are assigned a substantial amount (>5%) of ancestry from a component (green) that is shared with individuals from the Levant_ChL population, and that is nearly absent in all other individuals in the Levant_N population. We therefore repeated these formal analyses of population substructure to be confident that our subsequent analyses about the ancestry of the Levant_ChL are not biased by any previously undetected substructure in the Levant_N population.

The Levant_N analysis population is comprised of 13 individuals from the Neolithic period in the Levant. One individual comes from the site Motza, in Israel, while the other 12 individuals come from ‘Ain Ghazal in Jordan. The sample from Motza is categorized as PPNB and those from ‘Ain Ghazal are categorized as both PPNB (n=10) and PPNC (n=2). Despite the geographic and temporal heterogeneity of these samples, formal symmetry testing indicates that they were consistent with being a homogeneous group relative to other Near Eastern populations to the limits of the resolution of the study in which they were first reported[1]. This genetic homogeneity is consistent with similarities observed in their material culture.

We repeated this formal symmetry testing analysis, further dividing the populations based on geography and time, and dividing them into three analysis populations:

(i) Motza (n=1)
(ii) ‘Ain Ghazal PPNB (n=10)
(iii) ‘Ain Ghazal PPNC (n=2)

We also separated the samples based on their ADMIXTURE results into two subsets:

(A) Levant Neolithic samples with <5% ancestry assigned to the green component (n=10)
(B) Levant Neolithic samples with >5% ancestry assigned to the green component (n=3)

See Supplementary Table 3 for individual subpopulation assignments.

Supplementary Table 3: Background and subpopulation groupings of Levant_N individuals.

			Subpopulation assignment	
I-ID	Site Location	Period	Geographic/Temporal	ADMIXTURE
I1679	‘Ain Ghazal, Jordan	PPNC	iii	B
I1416	‘Ain Ghazal, Jordan	PPNB	ii	A
I1415	‘Ain Ghazal, Jordan	PPNB	ii	B
I1414	‘Ain Ghazal, Jordan	PPNB	ii	A
I1701	‘Ain Ghazal, Jordan	PPNB	ii	B
I1709	‘Ain Ghazal, Jordan	PPNB	ii	A
I1727	‘Ain Ghazal, Jordan	PPNB	ii	A
I1710	‘Ain Ghazal, Jordan	PPNB	ii	A
I1707	‘Ain Ghazal, Jordan	PPNB	ii	A
I1704	‘Ain Ghazal, Jordan	PPNB	ii	A
I1700	‘Ain Ghazal, Jordan	PPNB	ii	A
I1699	‘Ain Ghazal, Jordan	PPNC	iii	A
I0867	Motza, Israel	PPNB	i	A

We computed symmetry statistics[2] of the form f_4(Levant_N_subset1, Levant_N_subset2; Test, chimp) for all combinations of subsets, for all ancient and present-day populations, *Test*. No *Test* contributed a Z-score ≥ |3| (see Supplementary Table 4), suggesting that it is appropriate to treat all subsets as a single homogenous population, as they are symmetrically related to all other *Test* populations after correcting for multiple hypothesis testing. We therefore conclude that our ability to model the ancestry of the Levant_ChL population is not impacted by any sort of hidden substructure within the Levant_N analysis population, and that it is best to analyze these individuals as a single population group in order to increase statistical power during subsequent analyses.

Supplementary Table 4: Statistics of the form f_4(Levant_N_subset1, Levant_N_subset2; Test, chimp) support grouping of all Neolithic Levantine individuals into a single analysis population.

Levant_N_subset1	Levant_N_subset2	Lowest z-score			Highest z-score		
		Test	Z	#SNPs	Test	Z	#SNPs
(i): Motza	(ii): ‘Ain Ghazal PPNB	EHG	-1.280	310463	Mota	2.500	320737
(i): Motza	(iii): ‘Ain Ghazal PPNC	Iran_HotuIIIb	-2.124	45422	Ju_hoan_North	1.814	236504
(ii): ‘Ain Ghazal PPNB	(iii): ‘Ain Ghazal PPNC	Iran_HotuIIIb	-2.143	30852	EHG	1.421	143667
A: <5% green	B: >5% green	Iran_HotuIIIb	-1.773	77511	Yoruba	1.985	409912

This table reports the Test populations that produced the highest and lowest z-scores for each sub-population combination. Z-scores ≥ |3| are considered statistically significant.

Supplementary Note 2 – *f*-statistics

We use *f*-statistics to formally test models for the relationship of the Levant_ChL population to other ancient Near Eastern populations. The process is described fully in the main text. Here we provide several diagrams outlining the tree-like relationships tested by the statistics, and discuss the meaning of the various results. For a full explanation of *f*-statistics, see Patterson et al.[2]

We start by testing whether a model in which the Levant_ChL population descends directly from the Levant_N population is plausible. We will refer to this as the "base model", and the following tree could be used to describe this relationship:

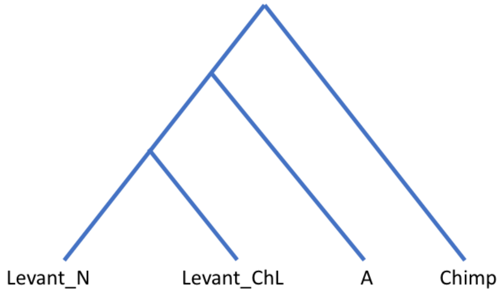

If the model is correct, this tree should be true for any population, A, in our dataset. Note that we use Chimp as the outgroup population in this tree, as we are confident that it is symmetrically related to all human populations.

Statistic 1: f_4 (Levant_ChL, A; Levant_N, Chimpanzee)

The first statistic we implement is used to determine whether Levant_N is more closely related to Levant_ChL than it is to any test population, *A*. This statistic can be thought of as testing an alternative tree from the base model described above. This alternative tree is of the form:

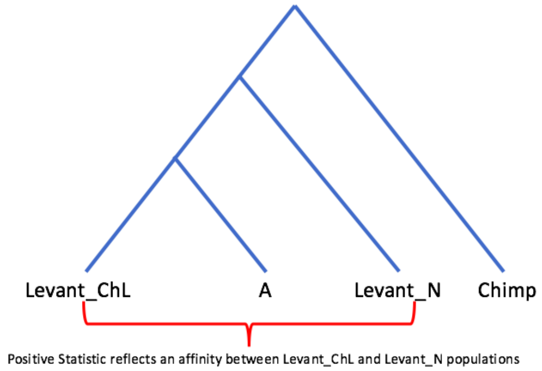

If the base model is true, we would observe a greater affinity between the Levant_ChL and Levant_N populations than expected by the alternative tree (highlighted by the red lines), and the statistic f_4 (Levant_ChL, A; Levant_N, Chimpanzee) would be positive. This is the case for every population, A, tested (see Figure 3a), so Levant_N and Levant_ChL are closely related.

Statistic 2: f_4 (Levant_N, Levant_ChL; *A*, Chimpanzee)

Next, we need to determine whether Levant_N and Levant_ChL form a true clade, meaning that they are symmetrically related to all other populations, *A*. This statistic can be thought of as testing a tree of the same form as the base model:

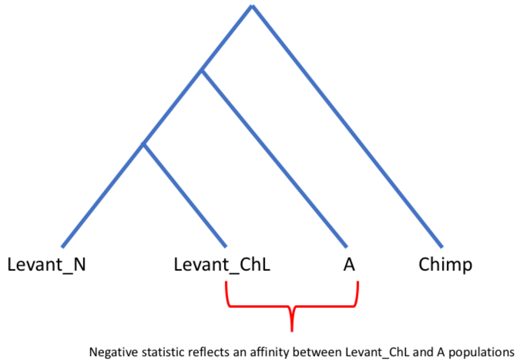

If the base model were true, the Levant_N and Levant_ChL should always be symmetrically related, relative to any other test populations, *A*, and the expected value of the statistic f_4(Levant_N, Levant_ChL; *A*, Chimpanzee) would be equal to 0. However, we observe a negative statistic in almost all cases (see Figure 3b), indicating that the Levant_ChL population must have ancestry related to population *A* that the Levant_N population does not have.

Considering these two statistics in combination, we conclude that the Levant_N and Levant_ChL populations are closely related (as indicated by statistic 1), but that they do not have a simple tree-like relationship (as indicated by statistic 2).

Statistic 3: f_3 (Levant_N, Levant_ChL; *A*, Chimpanzee)

One possible explaination for how we might explain the relationship between Levant_ChL and Levant_N is if the Levant_ChL population is admixed, with some of its ancestry coming from a population related to Levant_N and some of its ancestry coming from a different population that is more closely related to some of the other Near Eastern populations that we tested. In order to determine whether this is the case, we use f_3-statistics, which formally test for admixture. If an f_3-statistic is negative, admixture must have occurred. We test for admixture using the statistic f_3(Levant_ChL; Levant_N, *A*), which can be thought of as testing a tree of the form:

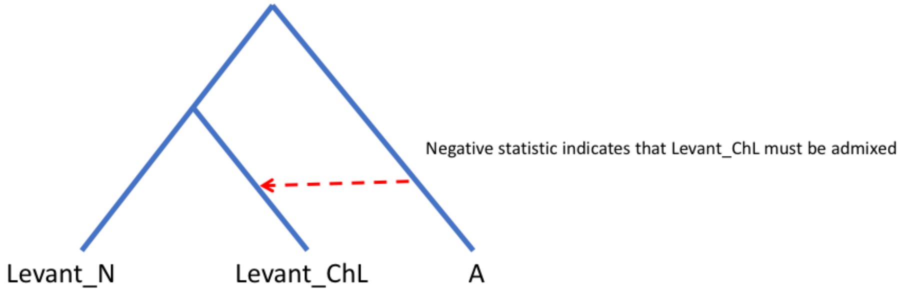

We find that the statistic is negative for many choices of *A* (see Figure 3c). Thus, the Levant_ChL population must be admixed. However, it is important to note that we cannot

assume that the tree that produces the most negative statistic represents the model that is closest to the truth. In order to identify the admixing populations, we must perform further tests.

Supplementary Note 3 – qpAdm Analyses

To identify the best source populations to model the ancestry of the Levant_ChL population, and to estimate their relative admixture proportions, we use the tool *qpAdm*. This method computes statistics of the form $f_4(\mathrm{Left}_i, \mathrm{Left}_j; \mathrm{Right}_k, \mathrm{Right}_l)$, for all possible pairs of populations in a proposed "Left" set and a proposed "Right" set of populations. The theory [3] shows that if the set of "Left" populations are descended from *N* ancestral populations that are related differentially to a set of "Right" populations, then the matrix of f_4-statistics will have rank *N-1*. We can compute a single P-value for a fit – fully taking into account correlation among neighboring positions in the genome and correlation in ancestry among populations – using a Hotelling's T-Test (empirically measuring the covariance of the matrix using a Block Jackknife). For a model that fits, we can then compute mixture proportions with appropriate standard errors.

qpAdm uses f_4-statistics to detect shared drift between the target population and the possible admixing source populations, relative to a set of differentially related outgroup populations. The target and admixture source populations are collectively referred to as "Left" populations, while the outgroup populations are collectively referred to as "Right" populations. The great advantage of *qpAdm* is that it does not require that the exact phylogenetic relationships between the target, admixture source and outgroup populations be known. We can visualize the approach using the following image:

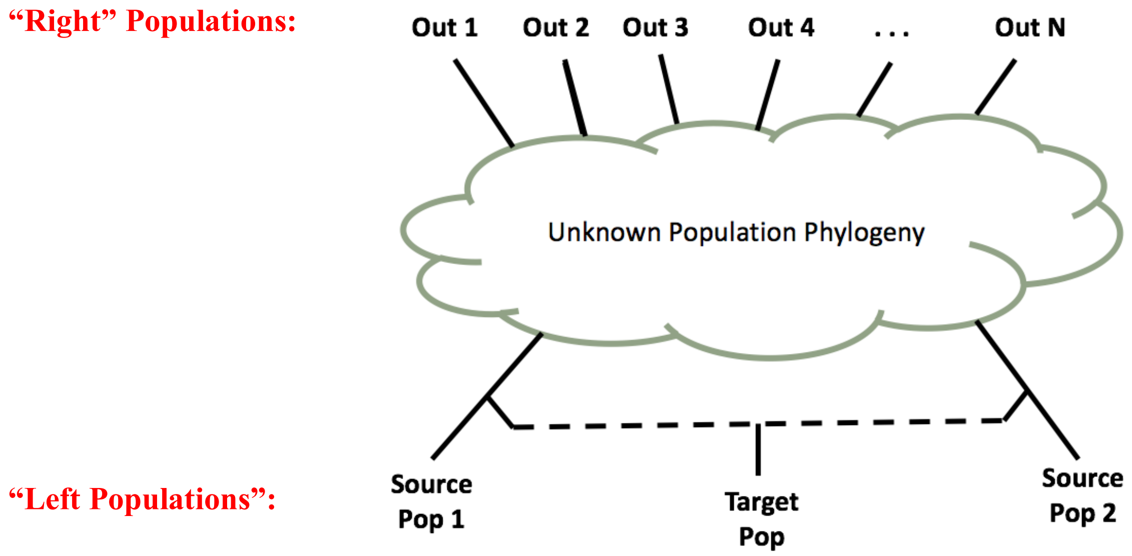

In what follows, we use this visualization to present the various models described in the text.

Model 1: No admixture

In the first analysis, we attempt to determine whether the Levant_ChL population can be modeled as being descended directly from the source population, relative to the outgroup populations. The model is tested using the 09NW set of "Right" population outgroups, and we used every available ancient population as a possible source population.

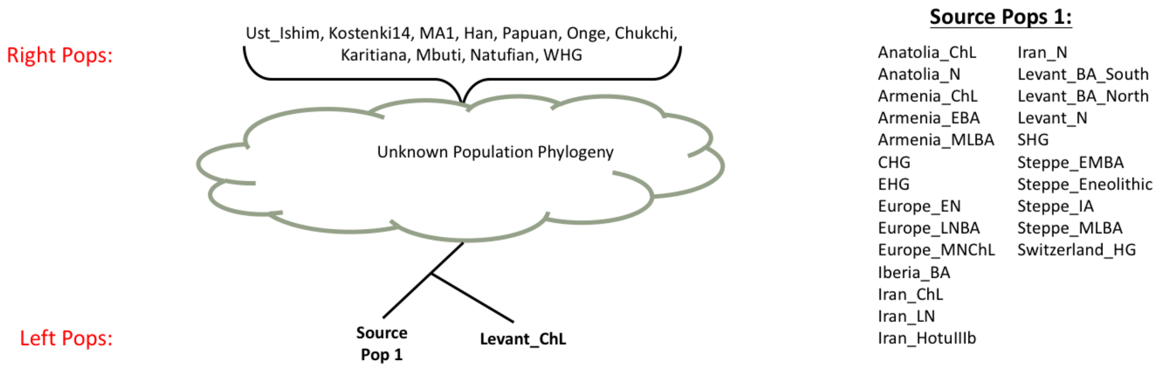

In every case, the model is rejected, with a p-value <0.05. These results are reported in Supplementary Data 2.

Model 2: Two-way admixture

In the second analysis, we attempt to determine whether the Levant_ChL population can be modeled as the product of a two-way admixture between two source populations. The model is tested using the 09NW set of "Right" population outgroups. To reduce the number of statistics calculated, we restrict the populations included as possible Source Pop 1 to the 6 populations that are most closely related to the Levant_ChL population in space and time. Every available ancient population was used as a possible Source Pop 2.

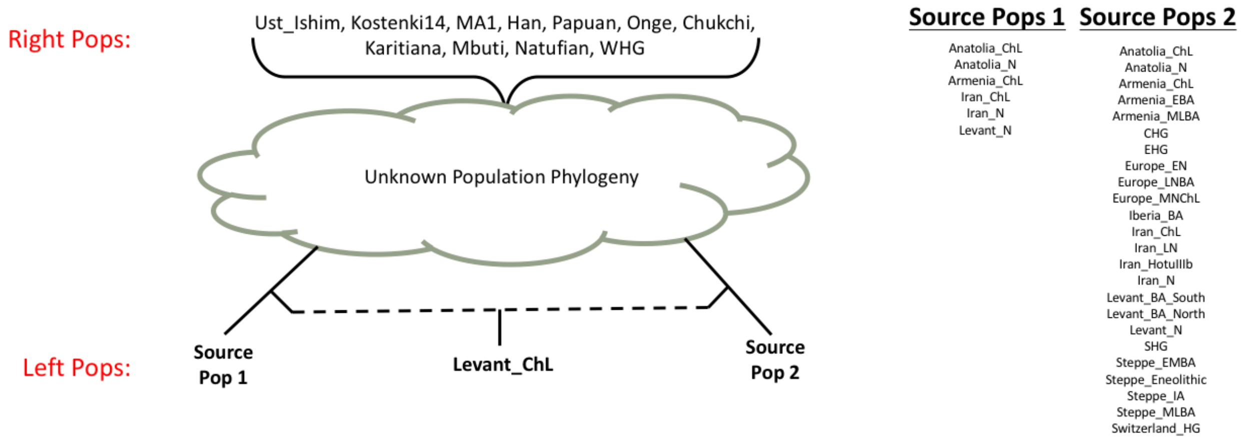

In every case, the model is rejected, with a p-value <0.05 or admixture proportions that do not fall within the bounds of 0-1. These results are reported visually in Supplementary Figure 2 and numerically in Supplementary Data 3.

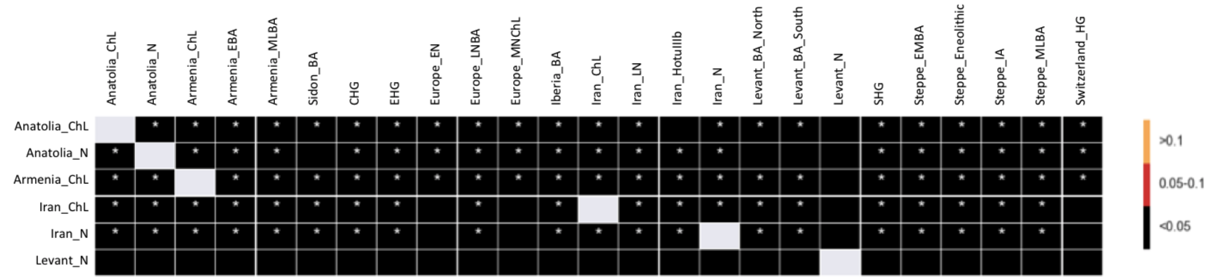

Supplementary Figure 2. Modeling Levant_ChL as a mixture of two populations, A and B. The heatmap presents qpAdm p-values for 2-way admixture models with 09NW "Right" population outgroups. Models with admixture estimates that do not fall within the bounds of 0-1 are considered implausible, and are colored black and marked with a white asterisk. Cases where the same population is used as both Source 1 and Source 2 are not part of this analysis and are shown in grey. There are no fitting models of two-way admixture.

Model 3: Three-way admixture

In the third analysis, we attempt to determine whether the Levant_ChL population can be modeled as the product of a three-way admixture between three source populations. The model is tested using the 09NW set of "Right" population outgroups. To reduce the number of statistics calculated, we restrict the populations included as possible Source Pop 1 and Source Pop 2 to the 6 populations that are most closely related to the Levant_ChL population in space and time. Every available ancient population was used as a possible Source Pop 3.

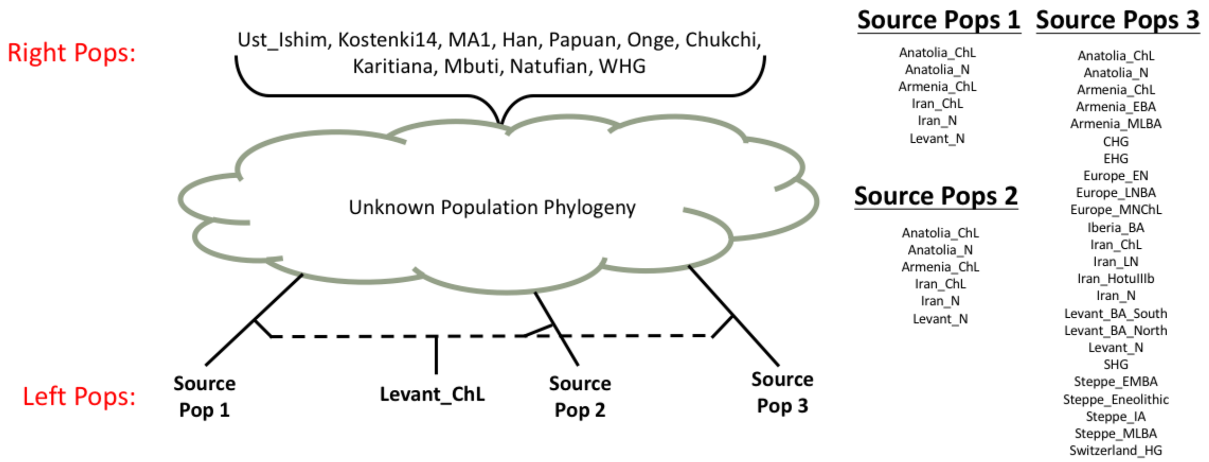

While most models are rejected, seven give p>0.05 results and have admixture proportions for all sources between 0 and 1. These seven models are reported in Table 1 of the main text. The full results are reported visually in Supplementary Figure 3 and numerically in Supplementary Data 4.

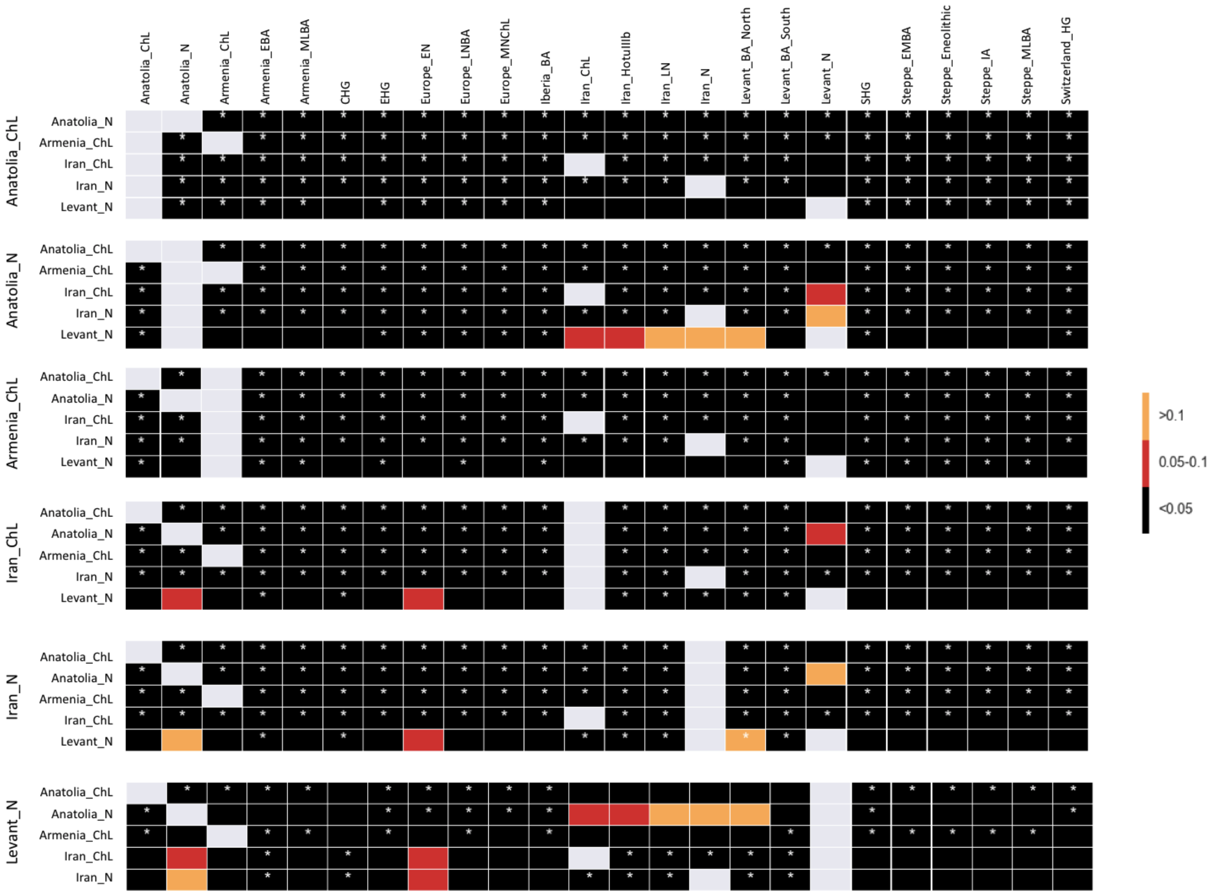

Supplementary Figure 3. Modeling Levant_ChL as a mixture of three populations, A, B, and C. The heatmap presents qpAdm p-values for 3-way admixture models with 09NW *Right* population outgroups. Models with admixture estimates that do not fall within the bounds of 0-1 are considered implausible, and are colored black and marked with a white asterisk. Redundant population combinations are shown in grey.

To distinguish between the seven plausible models, we add additional outgroups to the “Right” population set. We choose these outgroups by identifying populations that are differentially related to the source populations in different models using f_4-statistics.

First, we attempt to distinguish between models that contincludeain either Anatolia_N or Europe_EN as source populations. We use a statistic of the form f_4(Anatolia_N, Europe_EN; A, Chimp) to identify populations that are differentially related to these two (Supplementary Figure 4a). The top seven such candidate populations are Switzerland_HG, SHG, EHG, Iberia_BA, Steppe_Eneolithic, Europe_MNChL, Europe_LNBA (we chose populations with either the most negative or positive f_4-statistics that were not already being used in the models).

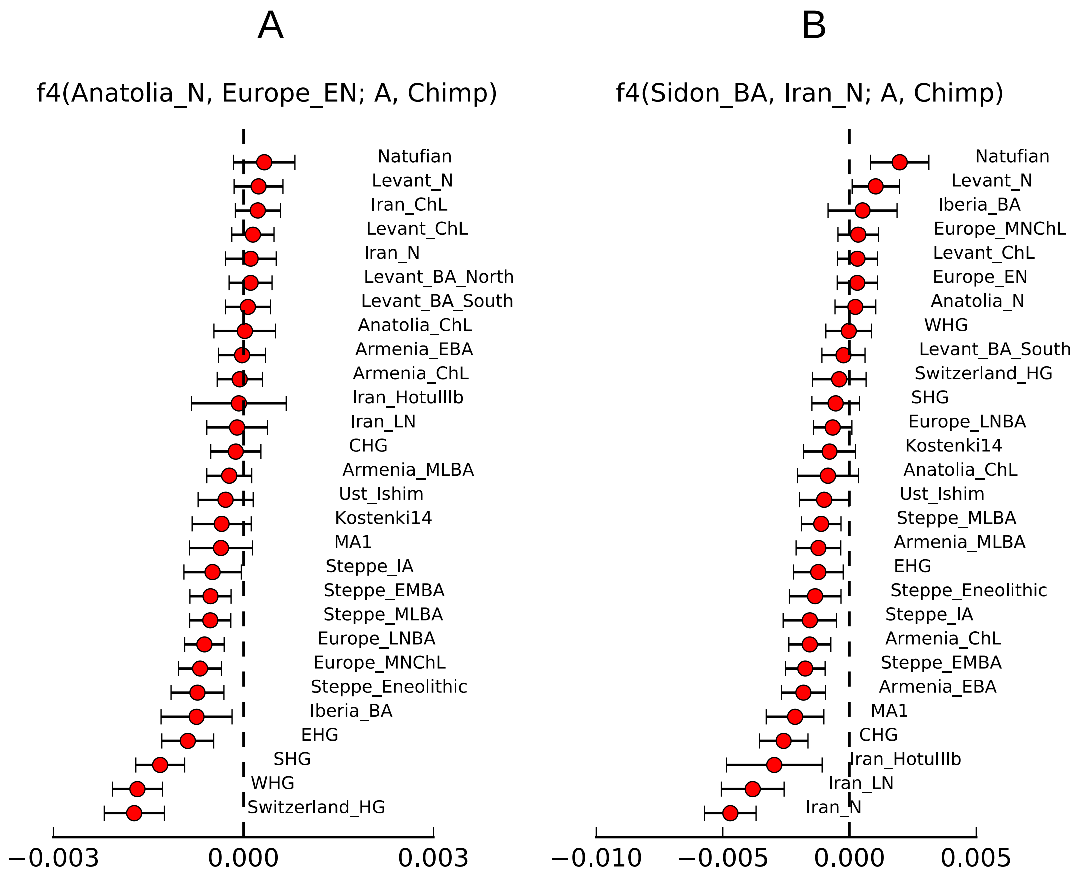

Supplementary Figure 4. f-statistics highlight differential relatedness between target populations and all ancient populations, A. (A) The statistic f_4(Anatolia_N, Europe_EN; A, Chimp) identifies populations that are differentially related to Anatolia_N and Europe_EN. (B) The statistic f_4(Levant_BA_North, Iran_N; A, Chimp) identifies populations that are differentially related to Levant_BA_North and Iran_N.

We then re-ran the three-way admixture model for all plausible models, adding one or more of the seven new candidate outgroup populations to the "Right" population set, and find that the addition of the Europe_LNBA population to the "Right" population outgroups eliminates all but two models. The two remaining models are (1) Levant_N, Anatolia_N, Iran_ChL and (2) Levant_N, Anatolia_N_Levant_BA_North (see Table 1 in the main text).

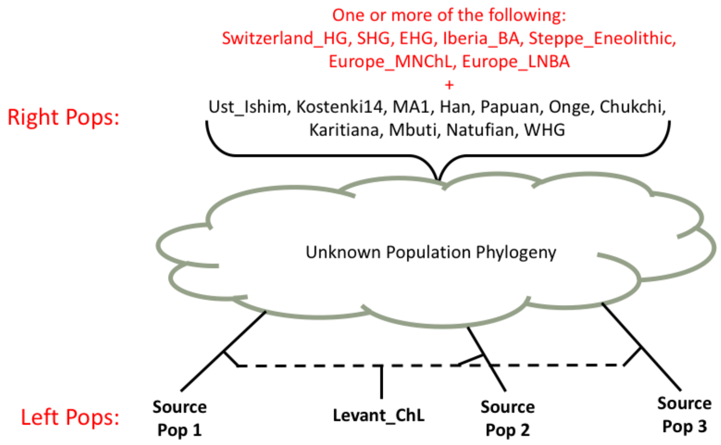

Source Pop 1	Source Pop 2	Source Pop 3
Levant_N	Anatolia_N	Iran_ChL
Levant_N	Anatolia_N	Iran_LN
Levant_N	Anatolia_N	Iran_HotuIIIb
Levant_N	Anatolia_N	Iran_N
Levant_N	Anatolia_N	Levant_BA_North
Levant_N	Europe_EN	Iran_ChL
Levant_N	Europe_EN	Iran_N

To distinguish between these two models, we use an f-statistic of the form f4(Levant_BA_N, Iran_N; A, Chimp), to identify populations differentially related to Levant_BA_N and Iran_N (Supplementary Figure 4b). The top candidate population is Iran_N (we chose this population because it had the most negative or positive f_4-statistic and was not already being used in the models). We then re-ran the three-way admixture model for the remaining plausible models, adding the Iran_N population to the "Right" population set containing 09NW and Europe_LNBA populations.

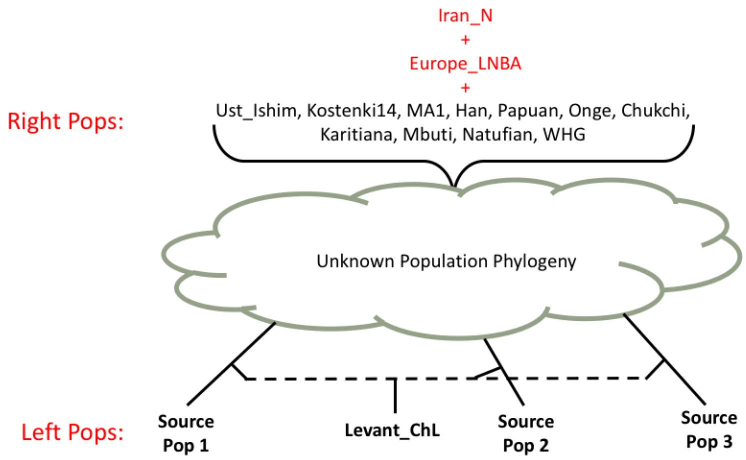

Source Pop 1	Source Pop 2	Source Pop 3
Levant_N	Anatolia_N	Iran_ChL
Levant_N	Anatolia_N	Levant_BA_North

This eliminated the model involving Levant_BA_North (see Table 1 in the main text). Finally, we reran *qpAdm* using all of the additional outgroup populations (Switzerland_HG, SHG, EHG, Iberia_BA, Steppe_Eneolithic, Europe_MNChL, Europe_LNBA, Iran_N) in the "Right" set, which produces a final estimate of the admixture proportions with the lowest standard error (see Table 1 in the main text).

Model 4:

We attempt to model the Levant_BA_North and Levant_BA_South populations as descended from Levant_ChL, to see if—with the addition of this new population—we have a better source

population than is available from the models proposed by Lazaridis et al. [3] and Haber et al. [4] which involved Levant_N and Iran_ChL.

First, we established that the Levant_BA_North and Levant_BA_South populations are two distinct populations, using the statistic f4(Levant_BA_North, Levant_BA_South; A, Chimp) (see Supplementary Data 5). We identify a number of cases in which the two populations are differentially related to a test population, A, and therefore conclude that the two populations and significantly distinct and should be treated separately.

Next, we model each population as the result of a two-way admixture between either Levant_ChL or Levant_N and some other source population, using the 09NW set of “Right” outgroup populations.

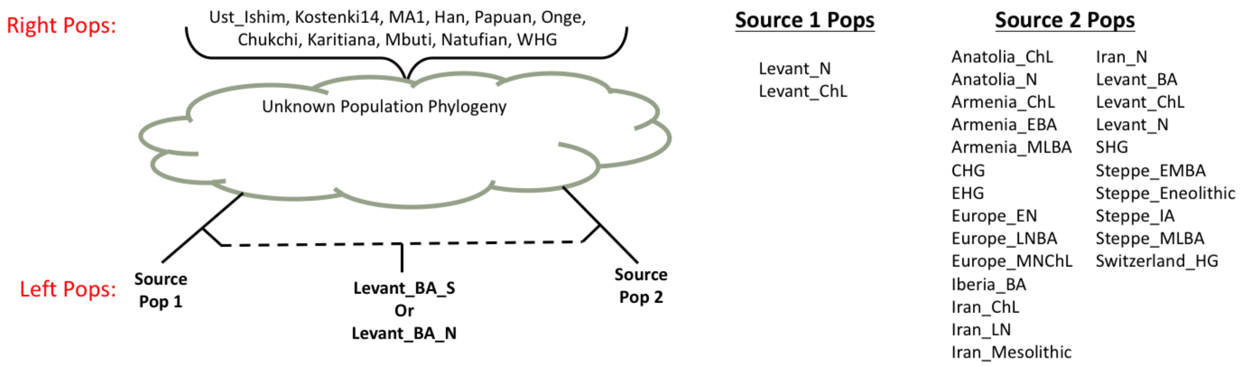

Several models are plausible in both cases (see Supplemental Figure 5 and Supplemental Data 6). Therefore, we returned to our procedure of identifying and adding additional population outgroups, and re-running the analysis.

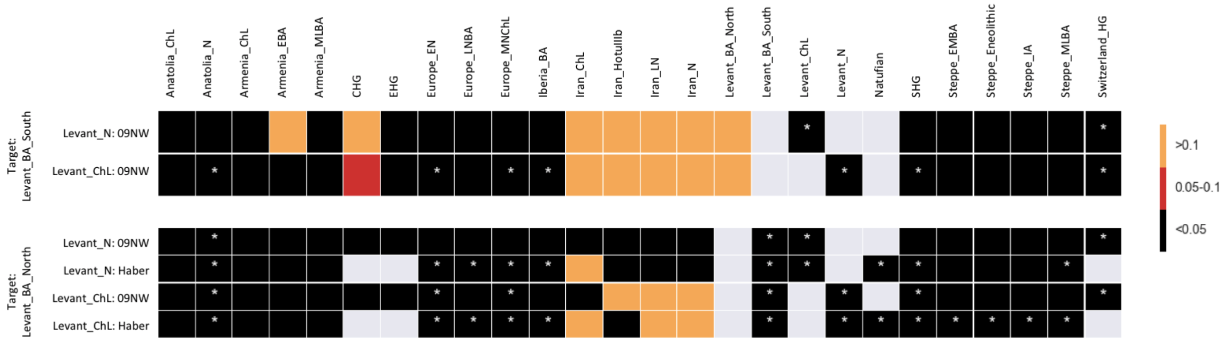

Supplementary Figure 5. Modeling Levant_BA_South and Levant_BA_North as mixtures of two populations, A and B. The heatmap presents qpAdm p-values for 2-way models describing target populations (top) Levant_BA_South and (bottom) Levant_BA_North with specified outgroups. Models with admixture estimates that do not fall within the bounds of 0-1 are considered implausible, and are colored black and marked with a white asterisk. Redundant population combinations or those with overlap between “Left” and “Right” populations are shown in grey.

In the case of Levant_BA_North, we repeat the analysis using the Right population outgroup set defined in Haber, et al. [4], and further examine the impact of the addition of the Anatolia_N population to this outgroup set. We find that the most robust model involves a two way admixture between Levant_ChL and either Iran_N or Iran_LN (see Table 2 in the main text).

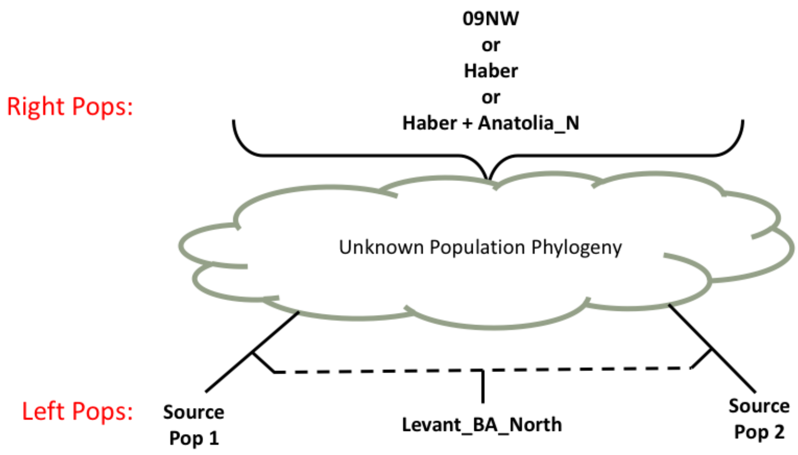

Source Pop 1	Source Pop 2
Levant_N	Iran_ChL
Levant_ChL	Iran_LN
Levant_ChL	Iran_N
Levant_ChL	Iran_HotuIIIb

In the case of Levant_BA_South, for models that appear plausible using the 09NW outgroups, we re-ran the analysis, adding various combinations of the following populations to the "Right" outgroup set: Armenia_EBA, Steppe_EMBA, Switzerland_HG, Iran_LN, and/or Iran_N. We find that the model (Levant_N + Iran_ChL) originally reported in Lazaridis et al. [3] remains the best model to describe the ancestry of Levant_BA_South—that is, the only one that is plausible among the models we tested.

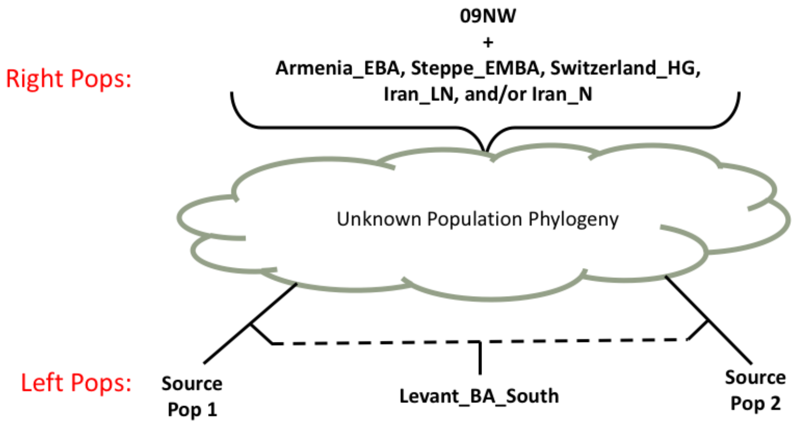

Source Pop 1	Source Pop 2
Levant_N	Iran_ChL
Levant_ChL	CHG
Levant_ChL	Iran_ChL
Levant_ChL	Iran_LN
Levant_ChL	Iran_HotuIIIb
Levant_ChL	Iran_N

Model 5

To distinguish between a model in which the most recent common ancestors of the Levant_ChL and Levant_BA_South populations split from one another before or after they split from their

shared admixture source populations used in the model (Levant_N, Iran_ChL), we added the Levant_ChL and Anatolia_N populations to the “Right” set of population outgroups.

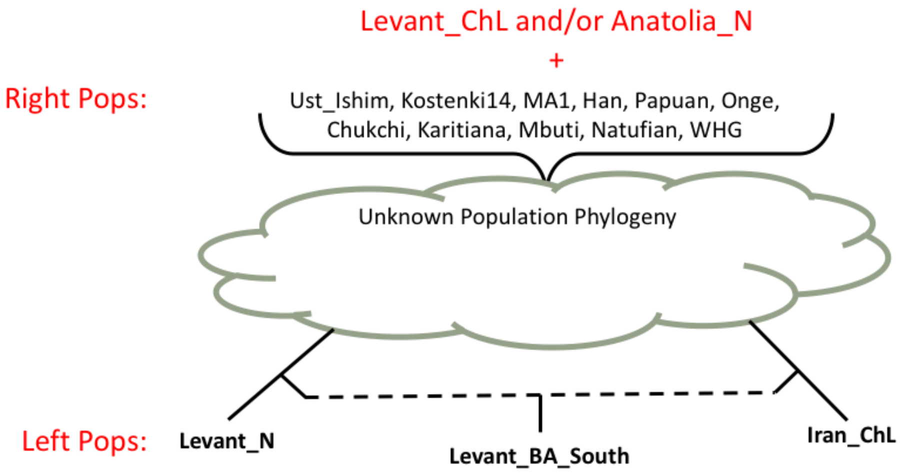

We find that the model fails when Levant_ChL and Anatolia_N are included in the “Right” population outgroups, suggesting that Levant_BA_South and Levant_ChL share drift that is not explained by their shared Levant_N and Iran_ChL ancestry.

Supplementary Note 4 – Population Admixture into East Africa from the Levant

West Eurasian ancestry detected in East Africans has been previously hypothesized to reflect a back-migration of West Eurasians into Africa[5,6]. In Lazaridis et al. [3], the authors systematically searched for a possible source of this admixture, modeling present-day East African populations as the product of an admixture event between populations related to the ancient Mota[7] individual and ancient Near Eastern populations. They conclude that Neolithic and Bronze Age Levantine populations serve as the best source of this ancestry. However, neither population fit as a model for all East African populations.

We now repeat this analysis, including Levant_ChL, to determine whether it may serve as a more proximal source of West Eurasian related ancestry in East Africa.

We performed *qpAdm* with the 08ENSW outgroups (Ust_Ishim, Kostenki14, MA1, Han, Papuan, Onge, Chukchi, Karitiana, EHG, Natufian, Switzerland_HG, WHG) used by Lazaridis et al. [3]. We attempt to model the East African populations, Luhya, Luo, Kikuyu, Jew_Ethiopian, Somali, Oromo, Masai, Dinka, Datog, Sandawe and Hadza, as a two-way admixture between the Mota individual, and all ancient Near Eastern populations in the dataset. Results are reported in Supplementary Data 8.

Lazaridis, et al. [3] find that Neolithic and Bronze Age populations from the Levant are the best single proxies for the source population of the West Eurasian related ancestry in East Africa, although neither population can serve as a source population for all East African populations. We find that the Levant_N and Levant_BA_South populations are both plausible sources for all but two of the East African populations (Oromo and Jew_Ethiopian). Levant_ChL is a plausible source population for all but three East African populations (Oromo, Jew_Ethiopian, and Masai). These results confirm that the West Eurasian ancestry observed in East Africa may be Levantine in origin, but suggest that the Levant_ChL population is not the best available source population to use to model this ancestry. Levant_N or Levant_BA_South are at least as good proxies for this ancestry, and possibly somewhat better.

Supplementary References

1 Lazaridis, I. *et al.* Genomic insights into the origin of farming in the ancient Near East. *Nature* **536,** 419-424 (2016).

2 Patterson, N. *et al.* Ancient admixture in human history. *Genetics* **192**, 1065-1093 (2012).

3 Haak, W. *et al.* Massive migration from the steppe was a source for Indo-European languages in Europe. *Nature* **522**, 207-211 (2015).

4 Haber, M. *et al.* Continuity and admixture in the last five millennia of Levantine history from ancient Canaanite and present-day Lebanese genome sequences. *bioRxiv*, 142448 (2017).

5 Pickrell, J. K. *et al.* Ancient west Eurasian ancestry in southern and eastern Africa. *Proceedings of the National Academy of Sciences* **111**, 2632-2637 (2014).

6 Pagani, L. *et al.* Ethiopian genetic diversity reveals linguistic stratification and complex influences on the Ethiopian gene pool. *The American journal of Human genetics* **91**, 83-96 (2012).

7 Llorente, M. G. *et al.* Ancient Ethiopian genome reveals extensive Eurasian admixture in Eastern Africa. *Science* **350**, 820-822 (2015).
