# Supplementary material for: Ancient DNA from Chalcolithic Israel reveals the role of population mixture in cultural transformation
Source: Nat Commun. 2018 Aug 20;9:3336. doi: 10.1038/s41467-018-05649-9 (PMC6102297; doi:10.1038/s41467-018-05649-9)
Supplement: Supplementary file 3 — Description of Additional Supplementary Files [file 41467_2018_5649_MOESM3_ESM.pdf]

Description of Additional Supplementary Files

File Name: Supplementary Data 1
Description: Library Information for All Samples Processed

File Name: Supplementary Data 2
Description: Modeling Levant_ChL as descended from a single source population

File Name: Supplementary Data 3
Description: Modeling Levant_ChL as descended from a mixture of two source populations

File Name: Supplementary Data 4
Description: Modeling Levant_ChL as descended from a mixture of three source populations

File Name: Supplementary Data 5
Description: Statistics of the form f_4(Levant_BA_North, Levant_BA_South; A, Chimp) test for cladedness between previously published Bronze Age groups from the Levant.

File Name: Supplementary Data 6
Description: Modeling Levant_BA_South and Levant_BA_North as descended from a mixture of two source populations

File Name: Supplementary Data 7
Description: Allele Frequencies

File Name: Supplementary Data 8
Description: Population Admixture into East Africa
